# New Method of Treating Ophthalmia*Read before the 30th Annual Meeting of the United States Veterinary Medical Association and First Veterinary Congress of America.

**Published:** 1893-11

**Authors:** R. H. Harrison


### NEW METHOD OF TREATING PERIODIC OPHTHALMIA BY SURGICAL INTERFERENCE (PARACENTECIS OF THE CORNEA), ILLUSTRATED BY CASES.*

By Dr. R. H. Harrison.

Every surgeon in the East, who has to do with animals brought to him for examination for soundness, is often very much annoyed, after giving a certificate, to have the animal brought back to him ater affected in one or both eyes with ophthalmia. Also, the treatment of these cases is unsatisfactory, for while the eye can be cleared up and brought apparently to a normal condition, the conscientious surgeon is obliged to tell the owner that the trouble is likely to recur and eventually end in blindness from cataract. The treatment recommended in the old, as well as the new, text books has been carefully and faithfully tried, and such meagre results obtained that I have been experimenting, and after treating over a hundred cases, have found a method of treatment, which at least is far more satisfactory in its results than by any other method laid down in the different text books. The different methods of treating the human eye and that of the horse are not comparative; for the veterinary surgeon who has studied and dissected minutely the eyes of both animals (human and equine), will observe great differences between them in a normal state; while again the habit and condition of both animals is widely different.

These experiments have extended over a period of eight years during my practice in Boston, and the West, and over one hundred animals, horses and mules, have been operated upon, and the result will be given.

Care should be used in every case that the conjunctival sac and the cornea be thoroughly examined, for although the membrana nictatans is very useful in removing foreign bodies from the cornea, bringing them towards the internal canthus so they may be washed away by the tears, sometimes the foreign body becomes imbedded in the cornea and the membrane cannot remove it, and straightway you have an inflammation of the cornea, in-

* Read before the 30th Annual Meeting of the United States Veterinary Medical Association and First Veterinary Congress of America.

volving the iris and adjacent tissues. In horses' eyes, which are as a rule brown, a magnifying lens is of much service in detecting foreign bodies.

*Tension* of the *eye-ball*—this is a useful and important guide in determining whether to operate or not, and in testing the eyes I have found the most practical way is to exert alternating pressure of two fingers placed on the upper lid, testing both eyes at once, using the index and middle fingers of both hands on the eyes. In this way a slight variation of the tension of the two eyes can be determined. If found harder than normal, the operation is indicated; but if softer, most emphatically contra-indicated, for surgical interference means an early and incurable blindness. It is a mistake to use the solution of atropia too long after the eye has been operated upon, as it causes too much photophobia, especially if the animal has to be worked during the day. It is well to remember that when one eye is affected with this disease that, as a rule, the other will become affected sooner or later; this should be especially remembered, for when you have one eye cleared up and apparently sound, a month or two later you are called to treat the other eye, affected more or less severely, either from sympathy, direct nerve influence, or by infection. The effects of cocaine appear to be especially beneficial in this operation; it renders the cornea and mucous membrane of the conjunctival sac and membrana nictatans non-sensitive to pressure, touch of the finger, or speculum. A great advantage from its use is its action on the small capillaries; they become for a time contracted and the operation is nearly bloodless. It also renders the use of fixation forceps unnecessary, as the eye-ball can be moved in any direction or desired position. A solution of cocaine, 5 per cent., is most advantageous. Discs of gelatine with cocaine are not satisfactory. The antiseptic solution to be preferred is boric acid, especially in treating the eye, as the bi-chloride of mercury causes the cornea to become too dry by continued use, and the epithelial layer is lifted up as a vesicle, and extensive and obstinate opacities may be formed. In using ointments or powders containing mercury, the administration of iodide of potassium internally is to be avoided, as this eventually reaches the tear fluid and the secretions of the conjunctiva, and changes it into the bin-iodide of mercury, which is very irritating and painful to the eye. Also calomel, if allowed to remain in the eye in too great quantities during the night, becomes changed into the bi-chloride of mercury, and acts as a caustic, and migth cause deep burns, followed by extensive sloughs of the conjunctiva, especially of the lower lid.

Cleanliness is very important before, during and after the operation. *Sponges are to be avoided*, and absorbent cotton substituted. Instruments are best disinfected by the heat and flame of an alcohol lamp. One of the best and cheapest antiseptic solutions to be used in washing away discharges, is chloride of sodium, a teaspoonful to the pint of water; it supplies a collyrium nearest in value to the natural secretion of the eye. If possible, I always advise an animal to be taken from work and placed in a well ventilated stall, darkened in front, and fed moderate rations of oats and hay, or, if in season, green stuff. *Corn* is to be *avoided*. If exercise seems to be necessary, have it done after sunset or on cloudy days. Excessive work, both draught and trotting, or running, should be limited.

The collyria used with the greatest advantage and success are:

Atropia sulphate, four grains to the ounce of distilled water. Cocaine, five per cent. solution of the hydro-chlorate.

Astringent Col.:

*R.* P. Zinci sulphatis, three grains.

Aq. lauro-cerasi, } Each, 3 ounces.

Aq. distillatæ, }

M. et. Ft. Col.

Mercurial Col.:

*R.* Ung Hydrg. sub. nit., thirty grains.
Al Amygdalæ, two drachms.
M. et. Ft. Col.

The most convenient method of applying collyrium to the eye, is by the Barnes eye dropper, or by a bit of absorbent cotton on the end of a match or tooth-pick. Especially in simple or complicated ophthalmia, bandages, ice-bags, sulphate of copper, local phlebotomy from the lachrymal vein are to be avoided, as they are too irritating and too severe in inexperienced hands. Also the applications of urine and the infusion of tea leaves, as these only complicate matters.

The use of the ophthalmoscope is very useful in making a diagnosis, also in ascertaining the condition of the lens after the operation has been performed and the eye cleared up; the normal reflex seems to be restored, although spots or specks are sometimes discovered on the anterior face of the lens, which if another attack does not take place, apparently remain in statu quo; and as far as I have observed rarely interfere with sight, except when large, then may make the animal shy, especially at reflected light from different objects.

I might note here, that the *healthy* eye should be studied and examined first, so that the normal appearance may be recognized as well as its differences in disease.

*Method of Operation.*—The patient is prepared by diet, quiet and darkness *immediately*. Collyrium of atropia used morning and night in *both* eyes, to properly dilate the pupil and prevent a hernia of the iris. If much photophobia is present use col. of boric acid, or chl. sodium, together with a two per cent. solution of cocaine. If much opacity, and a tendency toward ulceration of the cornea, the collyrium of mercury is indicated. Continue this treatment form 36 to 48 hours, then confine your animal for operation, using a twitch, while an assistant holds the ear with one hand and the nose above the false nostril with the other. Make application of the five per cent. solution of cocaine to both eyes, by means of absorbent cotton, taking pains to render insensible the whole lachrymal sac, and especially the membrana nictatans, this generally requires from five to ten minutes. When the eye is insensible to the touch of the finger, the self-retaining eye speculum is introduced, and with the narrow cataract knife of Van Graefe's, modified by having it only half the usual length of the blade, make an incision, or puncture, at the lower margin of the cornea and sclera, midway between the external and internal canthi; this can be expedited by slight pressure on the cornea (previously oiled with almond oil). By allowing the point of the knife to remain in the wound the aqueous humor will escape. I allow enough to come out so that the cornea has a flattened appearance. Care must be exercised that the flow of aqueous is very gradual, or the lens might be torn from its attachments, the iris also involved. The other eye is operated upon in the same manner, only less aqueous humor is allowed to flow out. This operation on an apparently sound eye, at the same time as the operation is performed on a diseased one, seems to be indicated by experience, for when only one eye is treated, the other, sooner or later, becomes involved.

The after treatment consists in the same rest, quiet and shade, together with an application of the atropia solution every second or third day, not oftener, or the animal will suffer too much from photophobia—also applications twice a day of the mercurial collyrium, until the opacity is removed. The salt and water solution is to be used daily, to remove discharges, and also allowed to run into the conjunctival sacs.

In operating *care* must be used in making the puncture, as a large opening may result in a disastrous fistula—also in directing of the knife in puncturing, use care so as not to wound the iris: this can be easily avoided by not entering the anterior chamber too deeply, and by directing the knife in the direction of the centre of the cornea. Complications may ensue of hernia of the iris, ulcer of the cornea, or anterior synechia, together with fistula and escape of the lens and vitreous humor already mentioned: these can be avoided by care, cleanliness and judgment in operating.

The following cases, I have arranged according to the time operated upon:

1886–90

| | Animal. | Age. | No. Op. | Times Affected. | Result. |
|---|---|---|---|---|---|
| 1 | B. M. | 6 | One. | Once. | Good. |
| 2 | " | 5 | " | " | " |
| 3 | B G. | 8 | " | Three years. | Fair. |
| 4 | G. G. | 8 | " | Two " | Good. |
| 5 | R. G. | 6 | " | One " | " |
| 6 | " | 6 | " | Once. | " |
| 7 | Bk. G. | 7 | Two | " | " |
| 8 | R M. | 10 | Three | " | Bad. |
| 9 | G. M. | 3 | One | " | Good. |
| 10 | " | 7 | " | " | " |
| 11 | B. S. | 4 | " | " | " |
| 12 | B. F. | 2 | " | From birth. | Bad. |
| 13 | G. F. | 3 | " | Once. | Good. |
| 14 | Bk. F. | 4 | " | " | " |
| 15 | R. F. | 2 | " | " | " |
| 16 | R. S. | 6 | " | Four years. | Bad. |
| 17 | Br. M. | 10 | Two. | Once. | Good. |

1891.

| | | Animal. | Age. | No. Op. | Times Affected. | Result. |
|---|---|---|---|---|---|---|
| | 1 | G. G. | 5 | One. | Once. | Good. |
| | 2 | Bk. S. | 12 | " | " | " |
| | 3 | C. M. | 5 | " | " | " |
| | 4 | Br G. | 5 | " | " | " |
| Twins. | 5 | G. G. | 7 | Two. | Every year. | Fair. Improved. |
| Twins. | 6 | " | 7 | Three. | " " | Bad. |
| | 7 | Bk G. | 9 | One. | Once. | Good. |
| | 8 | G. S. | 4 | " | Two years. | " |
| | 9 | G. G. | 3 | " | Once. | " |
| | 10 | B. G | 4 | " | " | " |
| | 11 | Bk. G. | 10 | " | 3 times a year for 4 years. | Bad. Shies badly. Small opacity of lens. |
| | 12 | G. G. | 7 | " | Once. | Good. |
| | 13 | B. G. | 5 | " | " | " |
| | 14 | G. G. | 6 | " | " | " |
| | 15 | Mule. | 3 | Two. | " | " |
| | 16 | B. M. | 8 | " | " | " |
| | 17 | Mule. | 4 | One. | Twice. | " |
| | 18 | " | 5 | " | 3 times a year for 2 years. | Bad. |
| | 19 | R. M. | 7 | " | Once. | Good. |
| | 20 | B. M. | 8 | " | " | " |
| | 21 | Mule. | Ag. | Two. | Every other month, 1 year | Improved. Fair. |
| | 22 | B. G. | " | One. | 1 year, 4 times | " |
| | 23 | Br. G. | 7 | " | Once. | Good. |
| | 24 | R. G. | 6 | " | Twice. | " |
| | 25 | R. M. | 6 | " | Once. | " |
| | 26 | Bk. G. | 5 | " | " | " |

1892.

| | Animal. | Age. | No. Op. | Times Affected. | Result. |
|---|---|---|---|---|---|
| 1 | Jack. | 4 | One. | Once. | Good. |
| 2 | Bk. G. | 8 | " | " | " |
| 3 | P'd. G. | 9 | Two. | Two years. | Improved. |
| 4 | P'd. M. | 8 | One. | Once. | Good. |
| 5 | Mule. | 4 | Two. | " | " |
| 6 | " | 5 | One. | " | " |
| 7 | B. G. | 6 | " | 3 times, 1 year | " |
| 8 | C. G. | 5 | " | Once. | " |
| 9 | G. M. | 8 | Two. | " | " |
| 10 | Bk. G. | 3 | One. | " | " |
| 11 | B. G. | 4 | " | " | " |
| 12 | B. M. | 4 | " | " | " |
| 13 | Bk. S. | 10 | Three. | Four years. | No more attacks. Bad results. Cataracts both eyes. |
| 14 | Bk. G. | 3 | One. | Once. | Good. |
| 15 | R. M. | 5 | " | " | " |
| 16 | R. G. | 7 | " | " | " |
| 17 | " | 9 | " | " | " |
| 18 | Mule. | 4 | Two. | Two years. | Improved. |
| 19 | Jennet. | 8 | One. | Once. | Good. |
| 20 | B. G. | Ag. | " | " | " |
| 21 | R. M. | " | " | " | " |
| 22 | B. G. | " | " | " | " |
| 23 | R. S. | " | Three. | Three years. | Bad. |
| 24 | G. S. | 4 | Two. | Two years. | " |
| 25 | R. M. | 5 | One. | Once. | Good. |
| 26 | Jack. | 4 | Two. | " | " |
| 27 | B. G. | 7 | One. | " | " |
| 28 | " | 12 | " | One year. | " |

1893.

| | Animal. | Age. | No. Op. | Times Affected. | Result. |
|---|---|---|---|---|---|
| 1 | B. G. | 6 | One. | Once. | Good. |
| 2 | B. G. | 7 | " | " | " |
| 3 | C. G. | 6 | " | " | " |
| 4 | C. G. | 5 | " | " | " |
| 5 | Mule. | 4 | " | " | " |
| 6 | C. S. | 8 | " | " | " |
| 7 | Br. G. | Ag. | Two. | Four years. | Bad. |
| 8 | Br. M. | 7 | One. | Once. | Good. |
| 9 | B. S. | 8 | Two. | Five years. | Bad. |
| 10 | B. M. | 4 | One. | Once. | Good. |
| 11 | G. M. | 6 | " | " | " |
| 12 | B. F. | 2 | " | " | " |
| 13 | B. C. | 1 | " | " | " |
| 14 | G. C. | 2 | " | " | " |
| 15 | Bk. G. | 4 | " | " | " |
| 16 | Bk. M. | 7 | Two. | Two years. | Bad. |
| 17 | Mule. | 5 | One. | Once. | Good. |
| 18 | " | 5 | " | " | " |
| 19 | R. M. | 4 | " | " | " |
| 20 | Shetl'd. | 3 | " | " | " |
| 21 | B. G. | 5 | " | " | " |
| 22 | C. G. | 4 | " | " | " |
| 23 | C. M. | 6 | " | " | " |
| 24 | " | 8 | Two. | Four years. | Improved. |
| 25 | R. S. | Ag. | " | Once. | Good. |
| 26 | R. G. | " | Three. | Five years. | Bad. |
| 27 | R. C. | 3 | One. | Once. | Good. |
| 28 | Br. G. | 5 | " | " | " |
| 29 | R. M. | Ag. | Two. | Four years. | Bad |

*Resumé.*—These cases, of which eighty per cent. have done well, ten per cent. an improvement and ten per cent. badly, have extended over a period of eight years. It should be borne in mind that the changeable nature of horse-flesh in regard to barter, should always be considered in giving statistics in the result of operations, and a surgeon's sucess may be limited to his knowledge of the case, as long as he can keep track of it. I would state that especially in the last three years, that in nearly every instance the case has been followed up as closely as possible. This has been much more easily done here, where horse flesh does not change hands as often as in the East. There have been within the last two years twenty-five animals that I have lost track of, as they were shipped to the Kansas City, St. Louis or Buffalo market, these I have not entered on the list.

